# Effect of Silica Size and Content on Superamphiphobic Properties of Silica-Fluoropolymer Core-Shell Coatings

**DOI:** 10.3390/polym12122864

**Published:** 2020-11-30

**Authors:** Jiyoung Lee, Ha Soo Hwang, Tien N. H. Lo, Won-Gun Koh, In Park

**Affiliations:** 1 Research Institute of Clean Manufacturing System, Korea Institute of Industrial Technology, 89 Yandaegiro-gil, Ipjang-myeon, Cheonan-si 31056, Korea; ttoittoijy@kitech.re.kr (J.L.); heliocity@naver.com (H.S.H.); htien@kitech.re.kr (T.N.H.L.); 2 Department of Chemical and Biomolecular Engineering, Yonsei University, 50 Yonsei-ro, Seodaemun-gu, Seoul 09722, Korea; 3 R&D Center, OomphChem Inc., 1223-24 Cheonan-daero, Seobuk-gu, Cheonan-si 31080, Korea; 4 KITECH School, University of Science and Technology (UST), 176 Gajeong-dong, Yuseong-gu, Daejeon 34113, Korea

**Keywords:** superoleophobic, superamphiphobic, fluoropolymer, core-shell, thiol-lactam initiated radical polymerization, spray coating

## Abstract

We present a facile approach to fabricate superamphiphobic surfaces by spray coating silica-fluoropolymer core-shell particles without substrate pretreatment with an additional binder resin. A series of SiO_2_@poly(1*H,*1*H,*2*H,*2*H*-heptadecafluorodecyl methacrylate) (SiO_2_@PFMA) core-shell particles with core particles of different sizes were prepared via thiol-lactam initiated radical polymerization (TLIRP). The surface of each SiO_2_ particle with an average particle size of 12, 80, 150, and 350 nm was modified with (3-mercaptopropyl) trimethoxysilane and used as a seed for TLIRP. The SiO_2_@PFMA particles with various SiO_2_ sizes and contents were coated on aluminum substrates by a spray gun and then thermally treated to form a stable, rough composite layer. During the spray coating, the core-shell particles were aggregated by rapid evaporation of the solvent and then irregularly adhered to the substrate resulting in hierarchical structures. In the case of SiO_2_@PFMAs with low SiO_2_ contents, the roughness created mainly by the polymer shell disappeared during heat treatment. However, the substrates coated with SiO_2_@PFMAs with high SiO_2_ contents maintained the roughness even after heat treatment. The core-shell particles prepared with 12 nm SiO_2_ formed a stable superamphiphobic surface. The water/hexadecane contact and sliding angles on an aluminum plate coated with SiO_2_@PFMA, prepared using 12 nm silica at 46 wt% silica content (12 nm-SiO_2_(46)@PFMA), were 178.5°/159.2° and 1°/7°, respectively. The cross-cut tape test showed that adhesion between the 12nm-SiO_2_(46)@PFMA and the aluminum substrate was classified as 5B. A glass surface spray-coated with the core-shell composite particles exhibited transparent superhydrophobicity and translucent superamphiphobicity by controlling the concentration of the coating solution.

## 1. Introduction

Over the past decade, superamphiphobic surfaces with both superhydrophobic and superoleophobic properties have drawn considerable interest in research and industrial applications [1,2,3,4,5]. Unlike superhydrophobic surfaces [6,7] that repel only water, superamphiphobic surfaces exhibit both water and oil contact angles exceeding 150° and sliding angles below 10° [8,9]. Compared with superhydrophobic surfaces, a superamphiphobic surface is more desirable in practical applications. For example, applying superhydrophobic technology to self-cleaning surfaces is effective in removing water-borne contamination, but the surfaces tend to be wet by oils rather easily. In contrast, oil- and water-borne contaminants bead up and readily roll-off from superamphiphobic surfaces. In addition, the application of superamphiphobic coating technology on the touch screen panel of smart devices can significantly reduce the occurrence of fingerprints and smudge deposition [10,11]. Thus, the superamphiphobic surface is useful for a variety of applications such as self-cleaning [12,13], anti-fouling [14], corrosion resistance [15], and anti-icing [16,17]. However, obtaining a superamphiphobic surface is challenging because organic liquids with low surface energy readily spread not only on most solid surfaces but also on superhydrophobic surfaces. To fabricate a superamphiphobic surface, it is necessary to develop a technology that combines elements such as low surface energy materials [18], fractal structure [19], and re-entrant features [8].

Numerous methods have been developed to create superamphiphobic surfaces using top-down methods such as lithography [5], etching [17,18,19,20], anodic oxidation [21], and laser processing [22,23], and bottom-up methods such as electro-spinning [24], nanofibers [25], sol-gel processes [26], particle casting [27,28], and spray deposition [29,30]. Top-down techniques (e.g., lithography) are useful for fundamental tasks such as the construction and design of superamphiphobic surface structures, but there are limitations in their application to commercial applications owing to the requirement of expensive equipment. Bottom-up methods (e.g., electrospinning) also have problems such as limitations of substrates, special equipment, harsh conditions, and low stability of the resulting surface roughness. Among the various methods mentioned above, the spray coating process is probably the most versatile coating technique and is particularly well suited for processes with high throughput and not limited to small areas. To fabricate a superamphiphobic surface by spray coating, a polymer-inorganic composite is the most useful material. Currently, various synthetic methods for polymer–inorganic composite materials that can be used to produce superamphiphobic surfaces via spray coating have been developed. In most cases, the superamphiphobic particles are prepared by modifying the surface of inorganic particles with a fluoro-alkoxy silane. The modified particles are sprayed directly onto a substrate pretreated with a binder [3] or mixed physically with other polymers (e.g., epoxy resin, fluoro-polymer) [1], which is then sprayed to create a superamphiphobic surface. However, although the technologies show good durability and high water/oil contact angles, the superamphiphobic particle itself lacks adhesion to the substrate, which entails cumbersome work such as pre-coating of the substrate, mixing with binders, and post coating of fluoropolymers.

On the other hand, a promising method for preparing a polymer-inorganic composite capable of spray coating is the fabrication of core-shell particles via surface-initiated polymerization (SIP) [29,30,31,32]. Using this method, a core-shell structure is formed using low-surface-energy fluoropolymers directly immobilized on the surface of inorganic cores. The core-shell structure can be dispersed in a spray process solvent, and the surface coated with it has many advantages such as roughness generated by inorganic particles, the durability of the composite, easy film formation, and good adhesion to substrates. Core-shell particles prepared by SIP have been reported for superhydrophobic surfaces, but the examples used for the fabrication of superamphiphobic surfaces are rare.

In our earlier study, we successfully produced a surface with high oleophobicity and superhydrophobicity by spray coating core-shell particles prepared by thiol-lactam initiated radical polymerization (TLIRP) [29]. Coating layers consisting of core-shell structures were produced through heat treatment after spraying and the wettability of the surface with roughness generated by the core SiO_2_ particles (372 nm size) were examined. However, despite its durability enhancement (40 cycles of tape peeling), good adhesion (5B), and superhydrophobicity (water contact/sliding angle = 164°/2°), the contact angle of hexadecane was 130.1°, its oleophobicity is not sufficiently high. To improve its oleophobicity, it is necessary to minimize the content of oleophilic segments in the polymer shell and further develop micro/nano roughness.

In this study, a series of SiO_2_@poly(1*H,*1*H,*2*H,*2*H*-heptadecafluorodecyl methacrylate (SiO_2_@PFMA) core-shell particles with different core sizes were prepared. After the heat treatment of the aluminum substrates coated with SiO_2_@PFMA particles, the micropapillae of the PFMA shell disappeared, and the roughness generated from the silica core assembly remained on the surface. We compared and analyzed the wettability of the surfaces depending on the particle size and the content of the silica cores. We also describe the transparent superhydrophobic and translucent superamphiphobic surfaces on glass slides by controlling the concentration of the coating solution.

## 2. Materials and Methods

### 2.1. Materials

Tetraethyl orthosilicate (TEOS, 98%), 1*H*,1*H*,2*H*,2*H*-heptadecafluorodecyl methacrylate (FMA, 97%), (3-mercaptopropyl)trimethoxysilane (MPTMS, 95%), tetrahydrofuran (THF, ≥99%), and α,α,α-trifluorotoluene (TFT, ≥99%) were purchased from Sigma-Aldrich (St. Louis, MO, USA). Aerosil 200 was purchased from Evonic (Piscataway, NJ, USA). The γ-Butyrolactam (BL, 99%), 1,1,1,3,3-pentafluorobutane (≥99.5%), and anhydrous toluene (99.8%) were purchased from Alfa Aesar (Ward Hill, MA, USA). Anhydrous ethanol (99.9%), ammonium hydroxide solution (NH_4_OH, 28.0–30.0%), and methanol (99.8%) were purchased from Samchun (Yeosu, Korea). The TFT and THF were distilled under argon before use. Aerosil 200 was dried in a dry oven at 100 °C before surface modification. FMA and BL were purified by passing the liquid substances through a neutral alumina column to remove the inhibitor prior to use. The 2, 2′- azobisisobutyronitrile (AIBN, 99%) (Sigma–Aldrich, St. Louis, MO, USA) was purified by recrystallization in methanol.

### 2.2. Synthesis of SiO_2_ Particles and Surface Modification (SiO_2_–SH)

SiO_2_ particles were synthesized according to a modified Stöber method [33]. Three different sizes of SiO_2_ particles were obtained by controlling the amount of reagents. Briefly, to synthesize SiO_2_ particles 80 nm in size, 68.15 mL of NH_4_OH (9 M) was added to 898.9 mL of anhydrous ethanol and the mixture was stirred for 10 min. Subsequently, 31.5 mL of TEOS was added to the solution and stirred for 24 h at 60 °C. The product was separated and washed three times with ethanol and twice with anhydrous toluene by centrifugation. Subsequently, the products were dispersed in 100 mL of anhydrous toluene using ultrasonication. For surface modification of the SiO_2_ particles, an excess of MPTMS was added to the dispersed solution and stirred for 24 h at 100 °C. After the reaction, the thiol-functionalized SiO_2_ particles (SiO_2_–SH) were washed several times with toluene and acetone by centrifugation and then dried in an oven overnight at 80 °C. To control the SiO_2_ particle size, 67.5 mL and 108 mL of NH_4_OH (9 M) were used to prepare SiO_2_ particles with sizes of 150 nm and 350 nm, respectively. Subsequently, the process followed was the same as the one mentioned above.

### 2.3. Synthesis of Core-Shell Particles by TLIRP

The FMA was polymerized from the SiO_2_-SH cores having various particle sizes. The method for the preparation of 12nm-SiO_2_(46)@PFMA with a silica content of 46 wt% is as follows; 0.5 g (theoretically 50 wt% of silica content) of 12 nm-SiO_2_-SH, 1 g of BL, and 1.5 g of TFT/THF mixture (80/20 wt%) were mixed using ultra-sonication and stirring, and then, 0.8 g of FMA was added to the mixture. A Teflon-coated stir bar was then placed in a 10 mL round flask equipped with a reflux condenser, and the flask was purged with argon gas and heated to 60 °C for 5 h. After polymerization, the flask was cooled to 25 °C, and the reaction mixture was precipitated in methanol. The product was washed and filtered with methanol and dried in a vacuum oven at 50 °C. Hereafter, the synthesized core-shell particles were designated as X-SiO_2_(Y)@PFMA and listed in Table 1, where X and Y indicate the SiO_2_ particle size and SiO_2_ content in the core-shell particles, respectively. In order to compare the thermal properties of the core-shell particles, the PFMA homopolymer was synthesized as follows. A 5 mL round bottom flask equipped with a stir bar was charged with 1 g of TFT, 1 g of FMA, and 0.01 g of AIBN. The flask was then purged with N_2_ gas and heated to 65 °C, and polymerization was conducted for 5 h. After polymerization, the reaction mixture was poured into methanol to precipitate the polymer and dried in a vacuum oven at 50 °C.

The conversion was calculated as follows;

(1) $$\frac{grafted\;polymer\;weight\;\left(A\right)\;}{feeded\;monomer\;weight\;\left(B\right)}*100=conversion\;\left(\%\right)$$

Herein, A is obtained by the below equation and C is the weight of the feeded SiO_2_–SH

(2) $$\frac{C}{C+A}*100=SiO_{2}\;content\left(\%\right)\;obtained\;by\;TGA.$$

### 2.4. Spray Coating of Core-Shell Particles

A series of SiO_2_@PFMAs were spray-coated onto aluminum plates for wettability measurements. Prior to spray coating, the aluminum plates were cleaned consecutively with water, acetone, and isopropyl alcohol. The cleaned substrates were then dried in an oven at 100 °C, and spray deposition processes were performed using a mixture of pentafluorobutane and THF as a solvent. A spray coating solution was prepared by adding 0.1 g of SiO_2_@PFMAs to 5 g of the solvent and dispersing it in an ultrasonic bath for 30 min. An airbrush was powered at a pressure of 20 psi. The distance between the airbrush nozzle and the substrate was maintained at 5–10 cm. After spray deposition, the coated Al plate was cured in an oven at 150 °C for 5 h. The same procedure as described above was also applied when the coating was performed using a glass slide as a substrate.

### 2.5. Characterization

Thermogravimetric analysis (TGA) measurements were performed at a heating rate of 10 °C/min from 30 to 700 °C under nitrogen purge with a TGA 4000 instrument (Perkin-Elmer, Foster City, CA, USA). Differential scanning calorimetry (DSC) analysis was carried out using a DSC 4000 instrument (Perkin-Elmer, Foster City, CA, USA). The following protocol was used for analysis: heating from 30 to 170 °C at a heating rate of 20 °C/min and cooling to −20 °C at a cooling rate of 20 °C/min, and then reheated to 170 °C at the same rate. The peaks for crystallization temperature (Tc) and melting temperature (Tm) were collected during the first cooling and second heating runs, respectively. XPS data were obtained using a K-alpha XPS system (Waltham, MA, Thermo Scientific, USA) with an Al Kα X-ray (1486.6 eV) source. Fourier transform infrared (FT-IR) spectra were recorded using Nicolet 6770 FT-IR Spectrometer (Thermo Scientific, Waltham, MA, USA) in the frequency range of 4000–400 cm^−1^. Scanning electron microscopy (SEM) was performed using a JSM 6701 instrument (JEOL, Tokyo, Japan) with a beam energy of 5 kV. Transmission electron microscopy (TEM) was performed using a drop of the dilute solution in trifluorotoluene on a carbon-deposited copper grid using a JEM-2100F instrument (JEOL, Tokyo, Japan) at an accelerating voltage of 200 kV. Contact angles were measured using the sessile drop method with a droplet of water or hexadecane at ambient temperature using a Smart Drop contact angle system (Femtobiomed, Gyeonggi-do, Korea). The cross-cut tape test was performed to investigate the adhesion properties based on the ASTM D3359 method. The coated substrate was scratched into 1 × 1 mm^2^ grids using a cut-off knife and peeled off with tape to measure substrate adhesion. A UV-vis spectrometer (Lambda 750S, Perkin-Elmer, Foster City, CA, USA) was used to obtain the transmittance of the coated glass plates in the 300–800 nm range.

## 3. Results and Discussion

**Figure 1:**
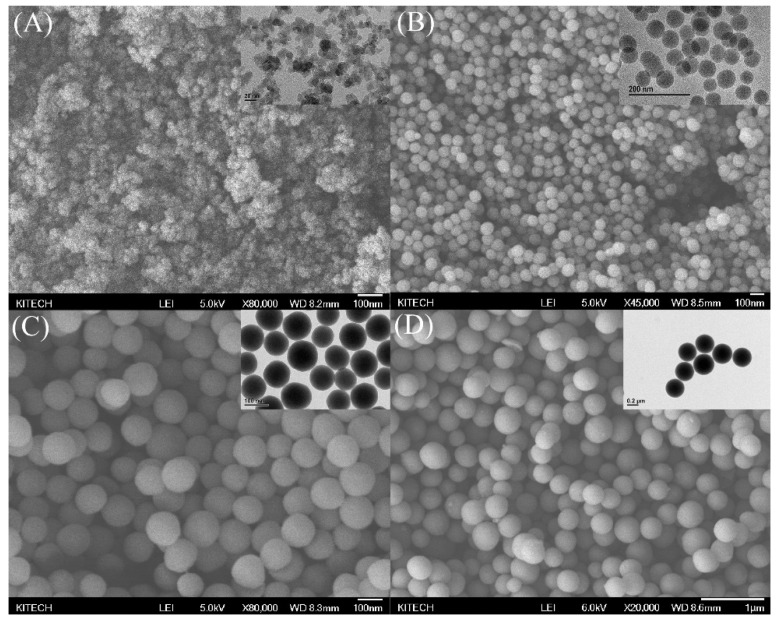
SEM images of (**A**) 12 nm-SiO_2_-SH, (**B**) 80 nm-SiO_2_-SH, (**C**) 150 nm-SiO_2_-SH, and (**D**) 350 nm-SiO_2_-SH (insets: corresponding TEM images).

SiO_2_@PFMA core-shell particles were synthesized using four silica particles with different average particle diameters of 12 (Aerosil 200), 80, 150, and 350 nm. By treating the SiO_2_ particles with MPTMS, a thiol-functional group capable of initiating the FMA monomer to form a polymer shell was introduced to each SiO_2_ particle surface. Figure 1 shows representative SEM and TEM (insets) images of the thiol-functionalized SiO_2_ (SiO_2_–SH) particles. Aggregation of particles is prevented by the stabilizing effect of the 3-mercaptopropyl group on the silica surface, and thus, all the SiO_2_ particles are well dispersed in THF [34]. As shown in Figure 1A, the 12 nm-SiO_2_-SH particles are irregularly shaped, and their average diameter is ~12 nm. In the case of 80 nm-, 150 nm-, and 350 nm-SiO_2_-SH particles synthesized using the Stöber method and surface-modified with 3-mercaptopropyl groups, the SEM images Figure 1B,C clearly confirm that the particles are monodispersed.

**Figure 2:**
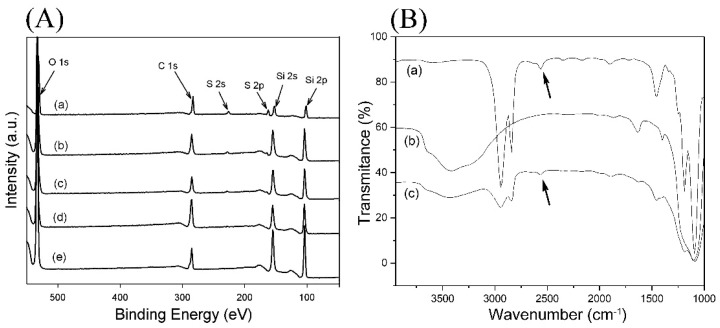
(**A**) XPS profiles of (a) 12 nm-SiO_2_-SH, (b) 80 nm-SiO_2_-SH, (c) 150 nm-SiO_2_-SH, (d) 350 nm-SiO_2_-SH, and (e) unmodified 12 nm-SiO_2_; (**B**) FT-IR spectra of (a) MPTMS, (b) 12 nm-SiO_2_, and (c) 12 nm-SiO_2_-SH.

The chemical composition of the SiO_2_–SH surface was investigated by XPS, as shown in Figure 2A. The XPS profile of the unmodified 12 nm-SiO_2_ shows peaks corresponding to Si2s (155 eV), Si2p (104 eV), and O1s (533 eV) (the C1s peak (285 eV) originating from the carbon tape used to support the specimen). In Figure 2Aa–d, the peak components at the binding energies of 228 and 165 eV are attributed to the S2s and S2p peaks, respectively, indicating the presence of the S–H group. In addition, the intensity of the S–H signal becomes stronger as the particle size decreases. As the particle size is smaller, its specific surface area is larger. As a result, the proportion of surface-covered S–H is relatively high. In the XPS profiles, the weight ratios of a, b, c, and d in Figure 2A are 1.5, 0.6, 0.4, and 0.2%, respectively. FT-IR analysis was performed to characterize the changes in the functional groups before and after modification of silica particles, and the IR spectra for 12 nm-SiO_2_ and 12 nm-SiO_2_-SH are shown in Figure 2B as representatives. In the spectrum of MPTMS (Figure 2Ba), the peak at 2570 cm^−1^ corresponds to the S–H stretching (the arrow in Figure 2B) and C–H stretching vibrations of the anchored propyl group appear at 2926/2855 cm^−1^ [34]. The characteristic peaks of S–H and C–H do not appear in 12 nm-SiO_2_ (Figure 2Bb), but they are clearly observed in the spectrum of 12 nm-SiO_2_-SH (Figure 2Bc), indicating that MPTMS reacts with the silanol groups on the SiO_2_ surface.

**Scheme 1:**
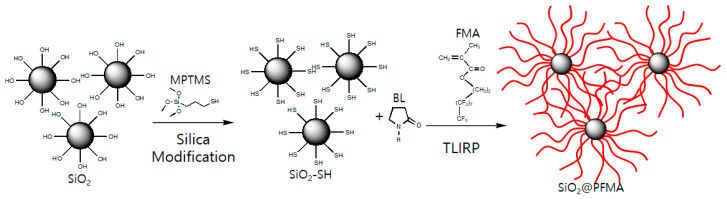
Synthesis of SiO_2_-SH and polymerization of FMA “*grafting from*” the SiO_2_-SH.

Each SiO_2_–SH particle is used as an initiator in the TLIRP system. Scheme 1 illustrates the general approach for the preparation of SiO_2_@PFMA core-shell particles. First, a thiol functional group is introduced through hydrolysis and condensation reactions between silanol groups on the surface of silica particles and MPTMS. PFMA chains are then grown from the SiO_2_–SH surface via TLIRP in the presence of BL. During mixing, the suspension of the SiO_2_–SH particles is opaque in the TFT/THF co-solvent. However, when BL is added, the dispersion of SiO_2_–SH is improved, and the liquid phase changes to be slightly translucent. This is because more complexes between SiO_2_-SH and BL are formed and the dispersion stability of the particles is improved in the reaction mixture. Finally, when the FMA monomer is injected, the entire liquid phase of the reactant becomes opaque because FMA acts as a non-solvent. Thereafter, as the polymerization proceeds, the reaction solution becomes increasingly cloudy, and in the final stage of polymerization, a high-viscosity polymer product is precipitated. The content of SiO_2_–SH in the core-shell particles is adjusted to ~25 wt% and ~45 wt% to compare the amphiphobicity of the coated composite layers in terms of the silica content.

Table 1 summarizes the silica contents, melting temperatures (*T*_m_), crystallization temperatures (*T*_c_), and monomer conversion of the SiO_2_@PFMA core-shell particles. The silica content of SiO_2_@PFMA particles is an excess of 3–11.7% relative to the feeding ratio of silica/FMA because the conversion rate of FMA is determined to be in the range of 58.4–76.6%. This conversion rate range is relatively low compared to that of SiO_2_@(PMMA-*co*-PFMA) in a previous study [29]. The solubility of the core-shell particles changes due to the growth of the PFMA shell, and thus they are precipitated during polymerization. This can also be explained by the co-solvent effect of the FMA monomer. In other words, at the beginning of polymerization, the liquid is mixed with other solvents and acts as a co-solvent to increase the solubility of PFMA. However, as FMA is gradually converted to PFMA, the relative concentration of FMA decreases, and the core-shell particles are precipitated. In addition, a reaction in the presence of smaller SiO_2_–SH particles exhibits a higher conversion rate of FMA because they have a higher content of the 3-mercaptoproply group due to the larger surface area of the silica. Therefore, when the same amount of particles is used, it has the same effect as using a relatively large amount of initiators [35].

**Figure 3:**
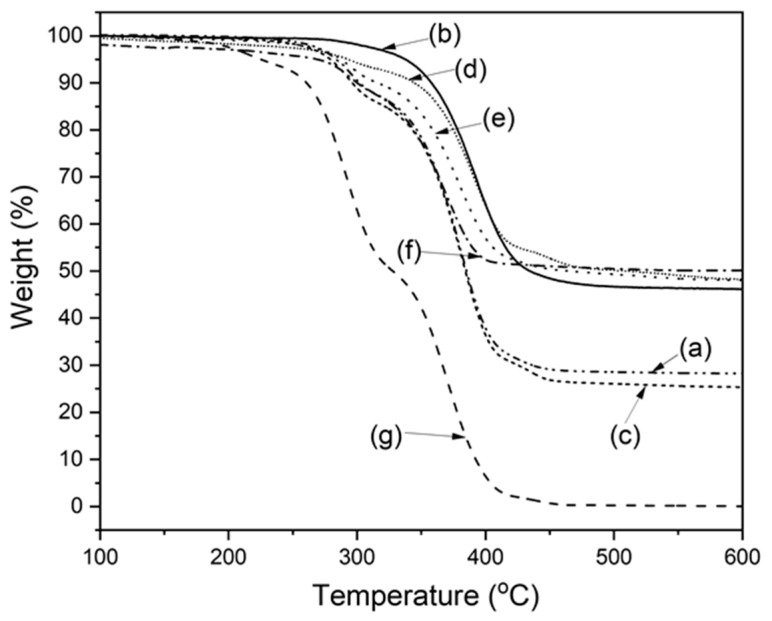
TGA curves of (a) 12 nm-SiO_2_(28)@PFMA, (b) 12 nm-SiO_2_(46)@PFMA, (c) 80 nm-SiO_2_(25)@PFMA, (d) 80 nm-SiO_2_ (49)@PFMA, (e) 150 nm-SiO_2_(47)@PFMA, (f) 350 nm-SiO_2_(52)@PFMA, and (g) PFMA homopolymer.

**Figure 4:**
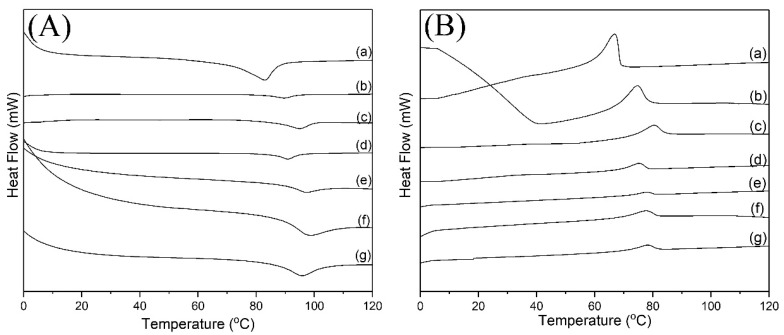
DSC curves of (**A**) 2nd heating and (**B**) 1st cooling; (a) PFDMA homopolymer, (b) 12 nm-SiO_2_(28)@PFMA, (c) 12 nm-SiO_2_(46)@PFMA, (d) 80 nm-SiO_2_(25)@PFMA, (e) 80 nm-SiO_2_(49)@PFMA, (f) 150 nm-SiO_2_ (47)@PFMA, and (g) 350 nm-SiO_2_(52)@PFMA.

The thermal properties of the SiO_2_@PFMAs were examined via TGA and DSC analyses. In Figure 3, SiO_2_@PFMA shows a major decomposition at 350–440 °C, indicating that the SiO_2_-SH surface is grafted by PFMA. The silica contents of the core-shell particles were calculated from the residue amounts obtained at 680 °C (Table 1). The phase transition temperatures were investigated by DSC analysis (Figure 4). In the case of the PFMA homopolymer, the *T*_m_ and *T*_c_ are 83.2 °C and 67.2 °C, respectively, similar to those reported in other studies [36,37]. SiO_2_@polymer core-shell particles exhibit a relatively higher phase transition temperature compared to that of the homopolymer constituting the shell because the polymer chain is immobilized on the SiO_2_ particles. The movement of the polymer chains immobilized on the particle surface is hindered, and more energy is required to cause a phase change [38,39]. For this reason, SiO_2_@PFMAs should show higher *T*_m_ and *T*_c_ values than those of the PFMA homopolymer. As expected, distinctly higher *T*_m_ (89.7–98.4 °C) and *T*_c_ (74.5–80.6 °C) are observed for all of the core-shell particles. It is also observed that a higher content of silica particles exhibit larger temperature values.

**Figure 5:**
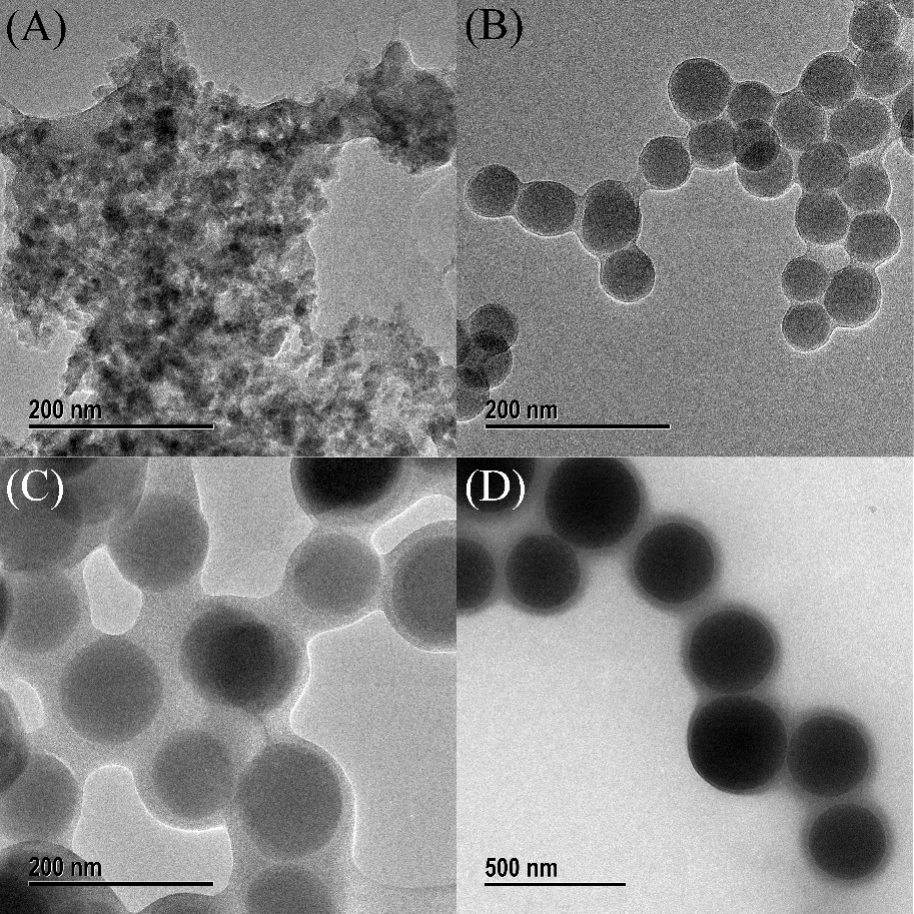
TEM images of (**A**) 12 nm-SiO_2_ (46)@PFMA, (**B**) 80 nm-SiO_2_(49)@PFMA, (**C**) 150 nm-SiO_2_(47)@PFMA, and (**D**) 350 nm-SiO_2_(52)@PFMA.

The morphologies and core-shell structures of the SiO_2_@PFMAs were observed by TEM, as shown in Figure 5. Unlike the TEM image of SiO_2_–SH (see Figure 1), a slightly dark-toned polymer film surrounding the dark SiO_2_ core is clearly observed. In Figure 5B–D, the spherical silica particles with different sizes are well separated at a certain distance, indicating that the PFMA shell is well-formed on the surface of each silica particle. In the case of 12 nm-SiO_2_@PFMA (Figure 5A), although it is not possible to specify a clear core-shell structure, a polymer shell is formed around the aggregate of irregular particles. From these observations, the SiO_2_@PFMA core-shell particles were successfully fabricated by TLIRP.

The SiO_2_@PFMAs were coated onto Al plates and the contact angles were measured to evaluate the influence of the SiO_2_ core size on wettability. Due to the low solubility of PFMA shells in organic solvents, a small amount of THF as a co-solvent was mixed with pentafluorobutane, a fluorine-based solvent, as a dispersion solvent for SiO_2_@PFMAs. The SiO_2_@PFMAs are well dispersed in the co-solvent and form a turbid suspension with a milky white color. Before spraying this solution, the surface of an aluminum plate was thoroughly cleaned to completely remove grime or oil as the presence of such substances can impede the adhesion of the spray plume. Our previous work showed that on spray coating a polymer solution or a dispersion of core-shell particles, random tiny droplets formed during the spraying process adhere to the surface of a substrate, providing the surface with the roughness of micropapillae. A superhydrophobic surface with a water contact angle higher than 150° can easily be fabricated because of the roughness created by submicron-sized polymer beads. However, this type of micropapillae roughness formed by the solid polymer can be easily damaged by physical touch, and therefore, superhydrophobicity can be easily lost. On the contrary, in the case of SiO_2_@polymer core-shell particles, the elastic behavior of the polymer chain becomes active, the irregular roughness becomes smooth, and the adhesion increases when the coated substrate is heated above the *T*_g_ or *T*_m_ of the polymer composing the shell.

**Figure 6:**
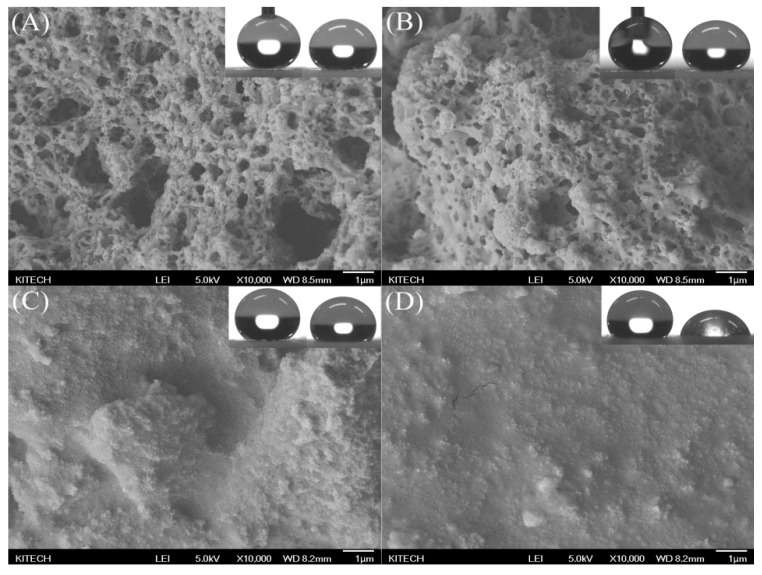
SEM images of spray-coated surfaces (**A**,**B**) before and (**C**,**D**) after heat treatment at 150 °C; (**A**–**C**) 12 nm-SiO_2_(28)@PFMA and (**B**–**D**) 80nm-SiO_2_(25)@PFMA.

To investigate the morphological changes induced by heat treatment of the surface, 12 nm-SiO_2_(28)@PFMA and 80 nm-SiO_2_(25)@PFMA were spray-coated on the Al plates and then thermally treated in an oven at 150 °C for 5 h. Figure 6 shows a top-view SEM image of the surface. The surface before heat treatment (Figure 6A,B) has a double-scale roughness structure in the form of irregular grains with a size of ~10 μm composed of submicron-sized grains. The formation of the hierarchical structures can be explained in terms of solvent evaporation and aggregation of the core-shell particles during the spray process. The aggregates of particles in the spray plume contain polymer covering several core-shell particles located in the center. These agglomerated particles adhere irregularly to the substrate surface, and the remaining solvent rapidly evaporates, forming numerous micropapillae. Therefore, the formation of irregular lumps is observed on the entire coated surface, but the shapes of the 12 nm and 80 nm SiO_2_ particles present in the core are rarely observed. The water- and oil-repellent properties of the coatings were examined by measuring the contact angles of water and hexadecane droplets on the surfaces. The water/hexadecane contact angles of the surfaces coated with the 12 nm-SiO_2_(28)@PFMA (insets of Figure 6A) and 80nm-SiO_2_(25)@PFMA (insets of Figure 6B) are 172°/164° and 174°/162°, respectively. The water sliding angles are <1° (near zero) on both the surfaces, while the hexadecane sliding angles are 14° and 15° for the surfaces coated with 12nm-SiO_2_(28)@PFMA and 80nm-SiO_2_(25)@PFMA, respectively. The results are in good agreement with those of other studies [30]. However, the core-shell particles exhibit almost the same wettability characteristics despite the different particle sizes. In other words, although it is a core-shell particle, the contact angles are more influenced by the roughness generated by the polymer shell. The roughness produced by the polymer shell on these surfaces disappears during the heat treatment. When the samples were treated at 150 °C (above the *T*_m_) for 5 h, the irregular roughness was leveled off and aggregates of silica particles in the form of small bumps are observed under the polymeric matrix. (Figure 6C,D) After the heat treatment of the surfaces coated with 12 nm-SiO_2_(28)@PFMA and 80 nm-SiO_2_(25)@PFMA, the contact angles for water/hexadecane are 144.4°/121.1° and 130.0°/82.5°, respectively. Smaller water/hexadecane contact angles than those of the untreated samples are observed for samples treated at 150 °C; this is attributed to roughness reduction on the top surface. Interestingly, the surface coated with 12 nm-SiO_2_(28)@PFMA exhibits higher water/hexadecane contact angles than those coated with 80 nm-SiO_2_(25)@PFMA because of the partially retained roughness produced by the 12 nm core silica particles even after heat treatment. The roughness of the coated surface after heat treatment depends on the size of the core particles, resulting in changes in the wettability.

**Figure 7:**
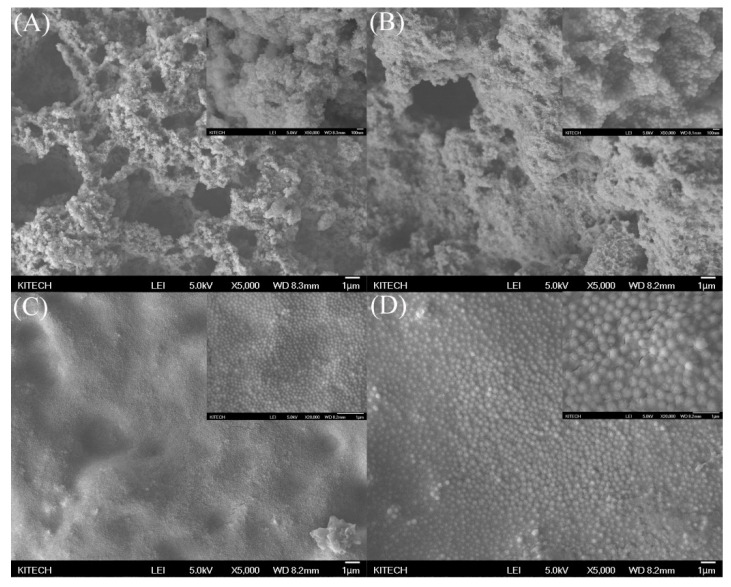
SEM images of spray-coated surfaces after heat treatment at 150 °C; (**A**) 12 nm-SiO_2_(46)@PFMA, (**B**) 80 nm-SiO_2_(49)@PFMA, (**C**) 150 nm-SiO_2_(47)@PFMA, and (**D**) 350 nm-SiO_2_(52)@PFMA.

To observe the effects on roughness as a function of core size, 12 nm-SiO_2_(46)@PFMA, 80 nm-SiO_2_(49)@PFMA, 150 nm-SiO_2_(47)@PFMA, and 350 nm-SiO_2_(52)@PFMA were coated on aluminum plates by spray coating. The coated plates were thermally treated at 150 °C for 5 h. Noticeable differences between the surface morphologies were observed for the samples prepared with various sizes of SiO_2_–SH. In Figure 7A,B when the silica content increases, the roughness of the surface is maintained even after heat treatment at 150 °C. However, in Figure 7C,D, low ridge shapes with silica particles are evenly dispersed on the flat surface. When the polymer shell containing similar silica content reaches the melting point, the phase transition proceeds more actively in SiO_2_@PFMA with a larger core size. In the case of core-shell particles with a larger specific surface area (smaller silica size), more surface roughness was maintained due to the limited movement of the particles. Consequently, the porosity and micropapillae generated during spray coating can be retained even after heat treatment. Maintaining such roughness after heat treatment is therefore a key factor influencing the formation of superamphiphobic surfaces.

**Figure 8:**
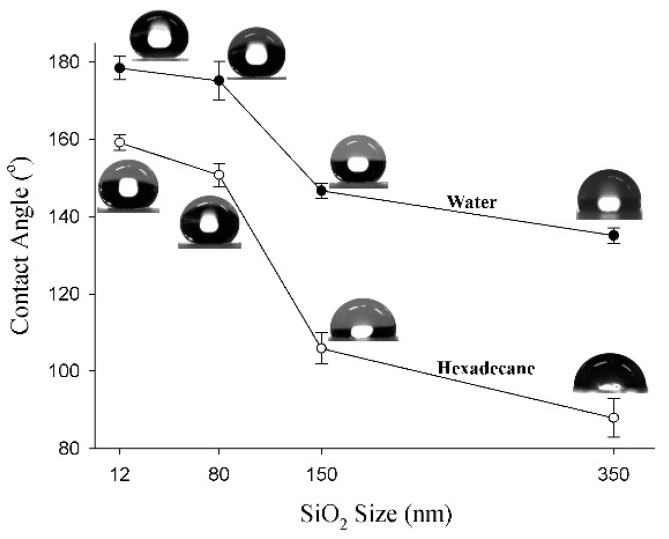
Water and hexadecane contact angles as a function of core SiO_2_ size.

Figure 8 illustrates the contact angles of water/hexadecane on the surface according to the size of the core SiO_2_ after heat treatment. The water/hexadecane contact angles of 350 nm-SiO_2_(52)@PFMA, 150 nm-SiO_2_(47)@PFMA, 80 nm-SiO_2_(49)@PFMA, and 12 nm-SiO_2_(46)@PFMA are 135.1°/87.9°, 146.7°/105.9°, 175.2°/150.8°, and 178.5°/159.2°, respectively. The contact angles for the samples with similar silica content (above 45 wt%) gradually increase as the size of the particles decrease. In particular, the water/hexadecane contact angles of 12 nm-SiO_2_(46)@PFMA and 80 nm-SiO_2_(49)@PFMA are ˃150°, which is indicative of superamphiphobicity. To be more specific, the hexadecane sliding angle of 12 nm-SiO_2_(46)@PFMA is less than 7°, whereas it is not measured for the 80 nm-SiO_2_(49)@PFMA because of the hexadecane droplet adhering to the surface. The water/hexadecane contact angles and the sliding angle of the 12 nm-SiO_2_(46)@PFMA surface before heat treatment are 174°/162° and 15°, respectively, similar to those of 12 nm-SiO_2_(28)@PFMA. As mentioned above, the roughness is produced mainly by the polymer shell. Therefore, it is not easy to confirm the influence of changes in the size or content of the core on the wettability before heat treatment. Furthermore, the change in the hexadecane contact and sliding angles is noticeable after heat treatment of the spray-coated 12 nm-SiO_2_(46)@PFMA surface. The contact angle for hexadecane is slightly lowered, while the sliding angle shows a significant improvement from 15° to 7°. These results cannot be explained by the change in roughness caused by heat treatment alone. In general, the nonpolar amphiphobic fluorinated moieties of FMA preferentially occupy the surface of the film, while the lipophilic groups of the PFMA backbone tend to face inward [40]. The orientation of more fluorinated moiety to the air increases a hexadecane sliding angle of the surface.

**Figure 9:**
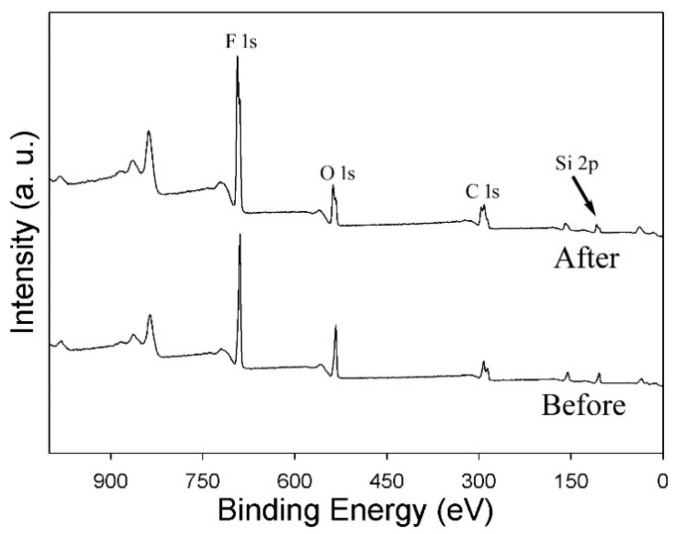
XPS profiles of the spray-coated 12 nm-SiO_2_(46)@PFMA surface before and after heat treatment at 150 °C.

**Figure 10:**
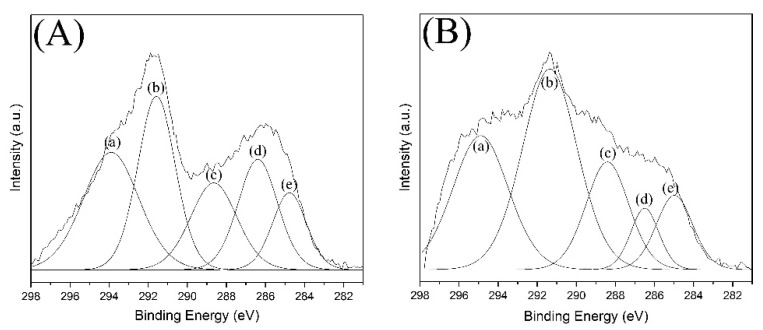
XPS C1s patterns of the spray-coated 12 nm-SiO_2_(46)@PFMA surface (**A**) before heat treatment and (**B**) after heat treatment at 150 °C.

XPS characterization was performed to determine the elemental compositions of spray-coated 12 nm-SiO_2_(46)@PFMA before and after heat treatment. The XPS wide-scan spectra before and after heat treatment of the 12 nm-SiO_2_(46)@PFMA surfaces are shown in Figure 9. Four element peaks including Si, C, O, and F are observed, and no significant difference between the spectra before and after heat treatment can be found. However, the F/C fraction increases slightly after heat treatment (Table 2). To obtain more detailed data, all C1s spectra were fitted using the five peaks arising from (a) CF_3_, (b) CF_2_, (c) C=O, (d) C–O, and (e) C–C, as shown in Figure 10. Peaks (c), (d), and (e) originate from the PFMA backbone. Peaks (a) and (b) correspond to the fluorinated side chains in the PFMA moiety. In Figure 10, the (c), (d), and (e) peaks are detected relatively strongly before heat treatment but are weaker than the (a) and (b) peaks after heat treatment. The lipophilic aliphatic group constituting the PFMA backbone is placed inside the film by heat treatment, and the fluoroalkyl chains faced the air. Due to the change in composition, the surface energy of the coating surface decreases, resulting in a low hexadecane sliding angle.

**Figure 11:**
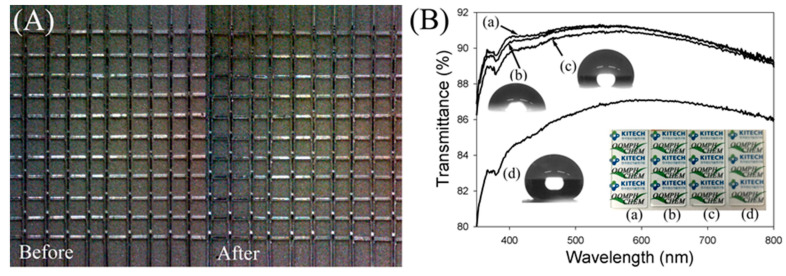
(**A**) Optical images of the cross-cut substrates before and after peeling off the tape and (**B**) transmittance spectra and optical images of glass slides spray-coated with 12 nm-SiO_2_(46)@PFMA with different concentrations; (a) bare glass slide, (b) 0.5 wt%, (c) 1 wt%, (d) 2 wt%. The insets are hexadecane droplet shapes for (b), (c), and (d).

The surface coated with 12 nm-SiO_2_(46)@PFMA was chosen to evaluate adhesion properties using the cross-cut tape test. Figure 11A shows photographs of the surface before and after the cross-cut tape test. The square edge showed no peeling marks and all of the squares remained intact without damage. According to the adhesion evaluation criteria, adhesion of the 12 nm-SiO_2_(46)@PFMA coating with the aluminum substrate was determined to be 5B.

In the case of the superamphiphobic surface, which was prepared by spray coating the core-shell particles, the roughness was obtained by the submicron-sized core-shell assembly. Such roughness is an important factor in controlling superhydrophobicity and superoleophobicity, but it is difficult to maintain transparency because the visible light gets scattered as a result of this. In previous studies, the transparency of the coating was achieved by diluting the concentration of the coating solution to reduce the coating thickness. However, if the concentration of a coating solution is overly diluted, the water- or/and oil-repellency of the coating is reduced due to insufficient coverage of the coating material on the substrate [30]. The particle layer formed by spray coating must possess a certain thickness in order to become a superamphiphobic surface. To investigate the transparency of the 12 nm-SiO_2_(46)@PFMA surface, spray coating was applied to the glass slides and the transmittance of light was measured. Figure 11B shows the variations in the transmittance profiles of the coated samples as a function of the coating solution concentration. The transmittances of glass slides coated with solutions of 0.5 and 1% concentrations are 91.1 and 90.8% at 550 nm, respectively. Compared to the transmittance of the original glass, it is slightly lower and almost transparent. The insets in Figure 11B are digital photographs of the coated glass slides overlaying printed paper. The print below is clearly seen through the glass slides coated with solutions of 0.5 and 1 wt% concentration. However, the surfaces are superhydrophiobic in nature and have water contact angles of ~170° but exhibit hexadecane contact angles of 90.6° (0.5 wt%) and 114.7° (1 wt%), to ensure that a superamphiphobic surface is not formed. When the concentration is increased to 2%, the transmittance rapidly decreases and the value is 86.9% at a wavelength of 550 nm. The printed image projected through the coated glass is relatively blurry (inset in Figure 11Bd) owing to the increased thickness and roughness of the coated layer, as described earlier [30]. The contact angles and sliding angles of water/hexadecane on this surface are 172.3°/155.6° and 8°, respectively. By controlling the concentration of 12 nm-SiO_2_(46)@PFMA, a nearly transparent superhydrophobic surface or an opaque superamphiphobic surface can be selectively prepared.

## 4. Conclusions

SiO_2_@PFMA core-shell particles were successfully synthesized via surface-initiated TLIRP in the presence of SiO_2_–SH and BL. The well-defined core-shell structures of the inorganic/organic hybrid particles were examined using DSC, TGA, SEM, and TEM analyses. SiO_2_@PFMA particles were spray-coated onto aluminum substrates to produce superamphiphobic surfaces, and they were thermally treated at temperatures above *T*_m_ to improve the olephobicity of the coated materials. No additional materials such as epoxy [1], PDMS [30], mortar [41], polyurethane [42], and PVT [3] were used to increase adhesion to the substrate. The spray-coated core-shell particles, with small sizes of silica particles, exhibited a hierarchical structure after thermal treatment, which was advantageous in the preparation of the superamphiphobic surface. On the surface coated with 12 nm-SiO_2_(46)@PFMA, the contact and sliding angles with respect to water/hexadecane were 178.5°/159.2° and 1°/7°, respectively, indicating the best superamphiphobic performance. When 12 nm-SiO_2_(46)@PFMA was coated on the glass surface, a transparent superhydrophobic surface could be prepared with a solution of below 1 wt% concentration, and a translucent superamphiphobic surface was formed using a solution of 2 wt% concentration.

## Figures and Tables

**Table 1:** Characterization of the SiO_2_@PFMA core-shell particles.

Sample Name	SiO_2_ Content (wt %) ^a^	T_c_ (°C) ^b^	T_m_ (°C) ^b^	Conversion (%) ^a^
12nm-SiO_2_(28)@PFMA	28.2	74.5	89.7	64.3
12nm-SiO_2_(46)@PFMA	46.2	80.6	95.1	72.8
80nm-SiO_2_(25)@PFMA	24.6	75.4	90.6	76.6
80nm-SiO_2_(49)@PFMA	48.7	78.2	97.5	65.8
150nm-SiO_2_(47)@PFMA	47.0	78.1	98.4	70.5
350nm-SiO_2_(52)@PFMA	51.7	78.2	95.9	58.4

^a^ calculated from TGA, ^b^ obtained by DSC.

**Table 2:** Element concentration of the spray-coated 12 nm-SiO_2_(46)@PFMA surface by XPS analysis.

Heat Treatment	Concentration (atom%)				
	Si 2p	C 1s	O 1s	F 1s	F/C
Before	9.48	27.97	20.18	42.37	1.52
After	7.52	28.09	17.19	47.20	1.68
